# INCREASE OF INCIDENCE OF CONGENITAL SYPHILIS IN SANTA CATARINA STATE BETWEEN 2007-2017: TEMPORAL TREND ANALYSIS

**DOI:** 10.1590/1984-0462/2020/38/2018390

**Published:** 2020-07-13

**Authors:** Julia Souza Vescovi, Fabiana Schuelter-Trevisol

**Affiliations:** a Universidade do Sul de Santa Catarina, Tubarão, SC, Brazil.

**Keywords:** Syphilis, congenital, Incidence, Pregnant women, Sexually transmitted diseases, Maternal and child health, Sífilis congênita, Incidência, Gestantes, Doenças sexualmente transmissíveis, Saúde materno-infantil

## Abstract

**Objective:**

To estimate the incidence of congenital syphilis and temporal trends of the reported cases of the disease in the state of Santa Catarina between 2007 and 2017.

**Methods:**

Observational study with retrospective cohort design, with secondary data from the Injury of Notification Information System (SINAN). Linear trend test and geoprocessing were performed to verify the behavior of the cases in the period.

**Results:**

There were 2,898 reported cases of congenital syphilis in the period, with an average of 2.9 per 1,000 live births in the period. There was an exponential increase of 0.9 percentage points per year, considered statistically significant (p<0.001). There was no difference between the incidences of cases in the different regions of the State. The fatality rate was 8.5%, considering deaths from syphilis, miscarriages and stillbirths. The profile was predominant of white mothers, with low schooling and 11.8% did not perform prenatal care. For this reason, 26.9% of them had a diagnosis of syphilis at the time of delivery. Most of the pregnant women (51.9%) had inadequate pharmacological treatment and 65.1% of the partners were not treated.

**Conclusions:**

There was an exponential increase tendency in cases of congenital syphilis in the State of Santa Catarina in the period studied in all regions of the State, which reveals the failure of prenatal care, late diagnosis and inadequate treatment of the pregnant woman and her partner.

## RESUMO

**Objetivo:**

Estimar a incidência de sífilis congênita e a tendência temporal dos casos notificados da doença no estado de Santa Catarina no período entre 2007 e 2017.

**Métodos:**

Estudo observacional com desenho de coorte retrospectiva, com dados secundários coletados no Sistema de Informação de Agravos de Notificação (SINAN). Foi realizado o teste de tendência linear e o geoprocessamento para verificar o comportamento dos casos no período.

**Resultados:**

No período, foram notificados 2.898 casos de sífilis congênita, com média de 2,9 a cada mil nascidos vivos. Houve crescimento exponencial de 0,9 ponto percentual ao ano, sendo estatisticamente significante (p<0,001). Não houve diferença entre a incidência de casos nas diferentes regiões do Estado. A taxa de letalidade foi de 8,5%, considerando os óbitos por sífilis, os abortos e os natimortos. O perfil predominante foi de mães da raça branca e com baixa escolaridade. Do total de mães analisadas, 11,8% não realizaram pré-natal - por esse motivo, 26,9% delas tiveram o diagnóstico de sífilis no momento do parto. A maioria das gestantes (51,9%) teve tratamento farmacológico inadequado e 65,1% dos parceiros não foram tratados.

**Conclusões:**

No período estudado, houve tendência de aumento exponencial dos casos de sífilis congênita em todas as regiões do Estado de Santa Catarina, o que revela a falha no pré-natal, o diagnóstico tardio e o tratamento inadequado da gestante e do seu parceiro.

## INTRODUCTION

Sexually transmitted infections (STIs) lead to economic, health and social problems, constituting a serious problem in public health, with a negative impact mainly on women and children.^1^ ^,^ ^2^ Syphilis has great epidemiological importance among STIs, considering the recent increase of its incidence as a risk factor for infection by the human immunodeficiency virus (HIV), in addition to the impact on the health of pregnant women and newborns with the increase in cases of congenital syphilis.^2^ ^,^ ^3^ ^,^ ^4^

Worldwide, approximately 2 million pregnant women are infected each year by *Treponema pallidum*, the etiologic agent of syphilis,^5^ and some of them do not receive the diagnosis during prenatal care. Among those diagnosed, there are cases of non-adherence to the indicated treatment, transmitting the infection to the fetus, who possibly will have the disease at birth, in addition to the risks of miscarriage, fetal death or sequelae caused by the infection.^6^ Moreover, reinfection may occur during pregnancy if the sexual partner does not receive adequate treatment, even if the pregnant woman has been treated, because previous syphilis does not confer immunity.^4^ There are also cases in which the treatment is performed inappropriately, such as in the 30 days before delivery and/or with incomplete therapeutic regimen, which can result in the ineffectiveness of pharmacological treatment.^7^

Given the difficulty of diagnosing the infection in asymptomatic children, diagnostic criteria of high sensitivity and low specificity are used:

- Every newborn, fetal death or miscarriage of a woman with untreated or untreated syphilis.
- Children under 13 with clinical manifestations, cerebrospinal fluid or radiological changes with a nontreponemic reagent test; children with results that react to nontreponemic tests (depending on the age group and the degree, as the case may be).
- Microbiological evidence of *T. pallidum* in biological samples or by direct microscopy.^8^

The analysis of the notified cases of congenital syphilis allows us to know the regional reality of the epidemic, as well as the factors associated to such incidence, in view of the decision making of public health managers regarding measures to cope with the scenario found. Considering the severity and epidemiological relevance of congenital syphilis, the present study aimed to analyze the temporal trend of cases notified between 2007 and 2017 in Santa Catarina State, with the objective of estimating the incidence of congenital syphilis and neonatal characteristics.

## METHOD

Observational study with retrospective cohort design. All reported cases of congenital syphilis (census) from 2007 to 2017 in Santa Catarina State were studied. Data were collected from the Information System for Notifiable Diseases (SINAN).^9^ For estimating incidence, the total number of live births in the period was investigated, distributed by year and municipality of residence.^10^

The database was obtained from the 20^th^ Health Management Event and the report of cases notified in the period, made available individually, in electronic banks with the extension .dbf. Data extraction took place in the SINAN database, with cases reported by congenital syphilis in the 295 municipalities in the nine macro-regions of Santa Catarina: Far West, Estuary of *Rio Itajaí*, Greater Florianópolis City, Midwest, North, West, *Serra Catarinense*, South and Itajaí Valley.

In order to perform the mapping of the analyzed phenomenon, the softwares Quantum GIS-QGIS^11^-and Microsoft Excel^®^ (2016) and the data tabulated by SINAN with occurrences identified by patient, month, year and municipality, among other data, were used. The use of Microsoft Excel^®^ (2016) enabled the creation of tables that totaled the cases per year by municipality.

The cartographic data used were the maps in shape format, in the official Brazilian cartographic system, of the municipalities and regional health centers based on the official data provided by the Brazilian Institute of Geography and Statistics (IBGE),^12^ as well as the annual population data of the same institute, published in the Brazilian Federal Official Gazette (*Diário Oficial da União* - DOU).^13^

With QGIS, the totalized tables of cases and the annualized population by municipality were linked, based on municipal codes; finally, the incidence of cases was calculated in the same environment of the Geographic Information System (*Sistema de Informação Geográfica* - GIS). each thousand live births, a variable that was mapped thematically, using the representation of classes by natural Jenks breaks from the most widely distributed map and maintained on the other maps, to allow comparisons and analysis of trends in the phenomenon, obtaining several maps. The statistical method of natural breaks by Jenks^14^ generates a defined set of thematic classes based on natural groupings according to the types of data that group similar values that maximize the differences between classes. Thus, the features are divided into classes in which there are relatively large differences in the data values, minimizing the sum of the variance within each class. This method is appropriate for mapping non-uniformly distributed values, just like the phenomenon under study in the present article.

Data on live births were obtained using the TABNET system of the Directorate of Epidemiological Surveillance of Santa Catarina State (*Diretoria de Vigilância Epidemiológica do Estado de Santa Catarina*),^15^ and statistical analysis was performed using SPSS software, version 21 (IBM, Armonk, New York, United States). For the presentation of data, descriptive epidemiology was used, with quantitative variables expressed in measures of central tendency and dispersion, and the qualitative variables in proportions.

This study was submitted to and approved by the Research Ethics Committee (CEP) on Human Beings at Universidade do Sul de Santa Catarina (Unisul) under Protocol Version 3, number 2.921.896, on September 27^th^, 2018.

## RESULTS

**Figure 1:**
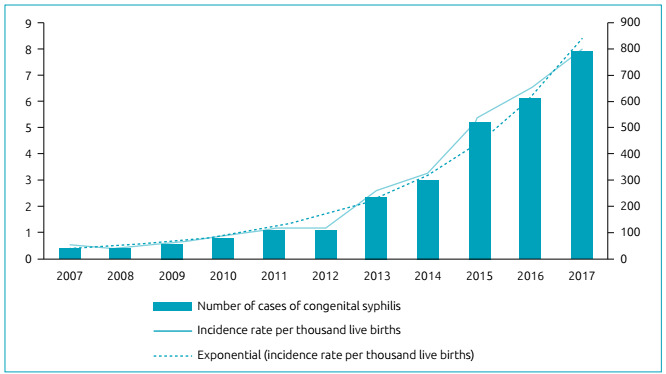
Temporal trend of the cases of congenital syphilis from 2007 to 2017 in Santa Catarina State.

During the study period, 2,898 cases of congenital syphilis were reported in a total of 985,985 live births in Santa Catarina State. The distribution of cases is shown in Table 1, and the rate of temporal trend in Figure 1. An exponential growth of 0.9 percentage points per year is observed, considered statistically significant (p <0.001).

**Table 1:** Distribution of notified congenital syphilis cases and number of live births in Santa Catarina State, from 2007 to 2017.

Year	Cases of congenital syphilis	Live births	Incidence rate per thousand live births
2007	41	82,170	0.5
2008	36	85,336	0.4
2009	54	83,563	0.6
2010	79	84,612	0.9
2011	109	87,483	1.2
2012	106	88,795	1.2
2013	240	89,899	2.7
2014	304	93,243	3.3
2015	522	97,231	5.4
2016	616	95,315	6.5
2017	791	98,338	8.0
Total	2,898	985,985	2.9

Source: Information System for Notifiable Diseases (SINAN), Directorate of Epidemiological Surveillance (DIVE)^9^.

**Figure 2:**
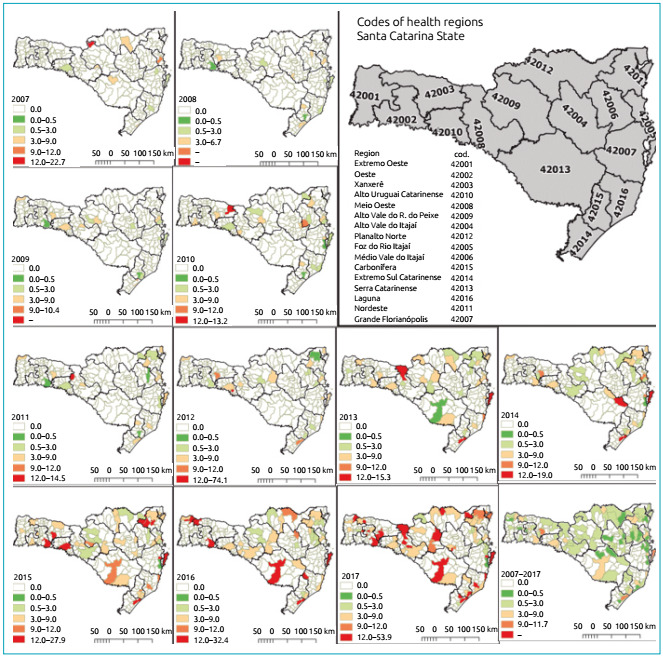
Annual incidence of congenital syphilis per thousand live births per year divided by macro-regions of Santa Catarina State, from 2007 to 2017.

The cases were distributed according to frequency per thousand live births in the State of Santa Catarina and divided by year, from 2007 to 2017. Figure 2 shows the temporal trend of incidence in the different geographical regions of Santa Catarina- no geographic pattern as to the distribution of cases was found, with homogeneity across the state. Nevertheless, the regions of *Planalto Norte* and *Serrano* deserve to be highlighted, because they had an important growth in the number of cases since 2014.

As for maternal sociodemographic characteristics, most were between 20 and 34 years old (the average age was 24.8±6.4, ranging from 13 to 45 years old), white complexion and had between five and eight years of schooling. Regarding clinical characteristics, most pregnant women underwent prenatal care, having been diagnosed with syphilis during this period. However, most of them did not undergo treatment for the disease or did it inappropriately, just as their partners did. As to neonates, most were white, diagnosed with syphilis at birth and, when present, the main symptom was jaundice. There was a slight predominance of the male gender. The main treatment regimen described was crystalline penicillin G, and the outcome was favorable in most cases. Table 2 presents the sociodemographic and clinical characteristics of the pregnant women, and Table 3, the characteristics of the children notified with congenital syphilis.

**Table 2:** Sociodemographic and clinical characteristics of pregnant women notified with congenital syphilis (n=2,898).

	n	%
Age in years old		
13-19	678	23.4
20-34	1,903	65.7
>34	275	9.5
Ignored	42	1.4
Ethnicity/Color		
White	2,325	80.2
Black	186	6.4
Asian	10	0.3
Pardo	315	10.9
Indigenous	28	1.0
Ignored	34	1.2
Education in years		
Cannot read	20	0.7
1-4	298	10.3
5-8	1,242	42.8
9-11	989	34.1
>11	109	3.8
Ignored	240	8.3
Prenatal		
Yes	2,485	85.8
No	342	11.8
Ignored	71	2.4
Moment of syphilis diagnosis		
Prenatal	1,894	65.4
Childbirth/curettage	779	26.9
After delivery	175	6.0
Not performed	11	0.4
Ignored	39	1.3
Treatment regimen for pregnant women		
Adequate	353	12.2
Inadequate	1,505	51.9
Not performed	923	31.9
Ignored	117	4.0
Partner (s) treated concomitantly with the pregnant woman		
Yes	776	26.8
No	1,888	65.1
Ignored	234	8.1

**Table 3:** Sociodemographic and clinical characteristics of children notified with congenital syphilis (n=2,898).

	n	%
Gender		
Male	1,389	47.9
Female	1,383	47.7
Ignored	126	4.4
Ethnicity/Color		
White	2,288	79.0
Black	108	3.7
Asian	9	0.3
Pardo	183	6.3
Indigenous	29	1.0
Ignored	281	9.7
Age of diagnostic (days)		
At birth	2,130	73.5
1-30	698	24.1
>30	70	2.4
Signs and symptoms (n=519)		
Jaundice	181	34.9
Rhinitis with bloody mucus	9	1.7
Anemia	63	12.1
Splenomegaly	55	10.6
Hepatomegaly	61	11.8
Osteochondritis	8	1.5
Skin lesions	56	10.8
Others	86	16.6
Treatment regimen		
Crystalline Penicillin G 10,000 to 150,000 IU/kg/day (10 days)	1,049	36.2
Penicillin G procraine 50,000 IU/kg/day (10 days)	439	15.2
Benzathine penicillin G 50,000 IU/kg/day	308	10.6
Another treatment regimen	589	20.3
Treatment not performed	420	14.5
Ignored	93	3.2
Evolution		
Alive	2,598	89.6
Death from syphilis	37	1.3
Deaths from other causes	17	0.6
Abortion	85	2.9
Stillborn	123	4.3
Ignored	38	1.3

The lethality rate for congenital syphilis was 8.5%, considering deaths from syphilis, abortions and fetal deaths. When considering only infant deaths from syphilis, the mortality rate was 3.6 per 100,000 live births.

## DISCUSSION

In order to verify the temporal trend of congenital syphilis in Santa Catarina State, the results of the present study demonstrated that there was an exponential growth of cases between 2007 and 2017. A possible cause of this increase is the improvement in the quality of data by SINAN.^15^ ^,^ ^16^ ^,^ ^17^ Nonetheless, when evaluating the cases of congenital syphilis and its characteristics, there is a great possibility that the increase in the incidence of the disease is the consequence of an inefficient prenatal care and the unavailability of raw material for the production of penicillin from 2014 (which led to the non-treatment of the disease or to alternative forms of treatment, such as cephalosporins),^18^ or the non-adoption of the recommended protocol, due to the high percentage of cases with inadequate treatment or non-treatment of the partner, once similar data are also observed in the rest of the country.^4^ ^,^ ^15^ ^,^ ^17^

According to the 2017 Syphilis Bulletin, by the Ministry of Health, the incidence rate of this disease had a progressive increase as of 2010. In 2016, the rate observed (6.8 cases/thousand live births) was three times higher than in 2006 (2.0 cases/thousand live births).^17^ Such rates are far from the national target: reduce to 0.5 case congenital syphilis per thousand live births until 2015.^18^ The bulletin also highlights that the increase was more expressive in Santa Catarina and Minas Gerais States, whereas Roraima, Amapá, Paraíba and Alagoas States showed a decrease in the incidence rate. In 2016, the Southern (7.7 cases/thousand live births), Southeastern (7.1 cases/thousand live births) and Northeastern (7.0 cases/thousand live births) regions had the highest incidences-even greater than the national rate. The Northern (5.4 cases/thousand live births) and Central-Western (4.8 cases/thousand live births) regions had lower rates than those of all Brazil, but they also increased when compared to previous years.^17^ In fact, data of the present study reveal that, in addition to the exponential growth in the number of cases of congenital syphilis, the highest rate was seen in 2017 in Santa Catarina State, higher than the national rate.

The incidence of congenital syphilis has also increased in other countries, such as the United States, which showed a considerable increase in rates over the same period.^6^ ^,^ ^19^ A recent systematic review concluded that screening for pregnant women infected with syphilis contributes to reducing the incidence of congenital syphilis.^20^ In 2016, the Ministry of Health implemented new strategic actions for the reduction of congenital syphilis in Brazil, thought precisely by the growing number of cases the country had been presenting. These strategies consisted of initiatives, such as effective prenatal care and adequate treatment for pregnant women and newborns who would present the disease.^21^ However, there was an increase in the absolute number of cases in 2016 and 2017, as well as the incidence of the disease, which shows the immediate ineffectiveness of the aforementioned initiatives.

Identifying the regions of Santa Catarina State with the highest incidence of the disease is essential to design control strategies. In the present study, a geographic pattern of the distribution of cases was not observed, but it was found that, between 2014 and 2017, there was an important increase in *Planalto Norte* and in *Serrano*. Thus, these are the regions that should receive greater attention from municipal and state managers, with the implementation of effective public policies that reduce the incidence of the disease.

As for congenital syphilis, the present study found a predominance of white people, followed by *pardo* people. National data show that syphilis is more common among black and *pardo* people. This difference can be explained by the fact that there is a predominance of white people in Santa Catarina due to the European colonization in the region.^22^ ^,^ ^23^ ^,^ ^24^

Low maternal education and the expressive percentage of pregnancy during adolescence are other factors associated to syphilis, found in Santa Catarina State and in the rest of Brazil.^17^ ^,^ ^18^ ^,^ ^24^ Moreover, it was found that, in most cases, treatment of the pregnant woman was not performed or was performed inappropriately, which also happened with their partners. The inadequate treatment of the partners, even when the pregnant women were treated properly, can lead to a reinfection of women by *T. pallidum*, with consequent infection of the newborn.^17^ ^,^ ^18^ An effective prenatal with information about syphilis, its early detection and its adequate treatment (both of pregnant women and their partners) are actions that can decrease the incidence of this disease.^17^ ^,^ ^18^ ^,^ ^25^

Nevertheless, a lack of raw material was reported for the production of benzathine penicillin to supply international shortages in 2014.^7^ The lack of medication and the epidemiological scenario that hit the country may possibly have led to an inadequate pharmacological treatment that, in turn, resulted in an increase in the rate of vertical transmission and an increase in neonatal morbidity and mortality.^6^ However, the lack of medication no longer affects Brazil. According to the 2017 Syphilis Bulletin, penicillin was prescribed in 88.9% of cases for treatment of syphilis during pregnancy in 2016.^17^ Even so, the number of cases of congenital syphilis has been growing, so the lack of penicillin should not be seen as its main causal factor.

There were many pregnant women who did not perform prenatal care, a fact that prevents the early diagnosis of the infection and, consequently, its treatment. In other regions of Brazil, there is also a tendency of not performing diagnostic screening tests, such as in Amazonas, Ceará, the Federal District, Espírito Santo, Rio de Janeiro and Rio Grande do Sul.^23^ The recommendation of the Ministry of Health is that serological screening during pregnancy should be performed using the rapid treponemal test in the first and third gestational trimesters and during hospitalization for delivery or curettage.^23^ In the case of pregnant women who have the reagent result, treatment and cure control should be carried out by titrating the Venereal Disease Research Laboratory (VDRL), which is a nontreponemal test.^23^ When observing such data, the hypothesis that the increase in the incidence of cases of congenital syphilis is strengthened is directly related to the difficulty of the health team in properly diagnosing and treating syphilis during pregnancy^1^ ^,^ ^18^-and one of the reasons may be the lack of preparation of these professionals to put into practice the protocols and recommendations of the Ministry of Health. Thus, the need for regularly training these teams is reinforced, with the objective of not only diagnosing the disease, but also accompanying and adequately treating these pregnant women.^5^ ^,^ ^16^

On the other hand, regarding the neonate, the diagnosis of the disease was made, in most cases, at birth, classifying it as early congenital syphilis. Such data is also found in other regions of Brazil: in a study carried out in the city of Sobral,^22^ for example, 88.2% of children were diagnosed with this disease within two days of life. Despite the early diagnosis, most children were asymptomatic, which highlights the importance of laboratory screening tests in the newborn whose mother was diagnosed with syphilis during pregnancy. When present, the most frequent symptom was jaundice, and the most common clinical manifestations of early disease are cutaneous and mucous membranes.^4^ ^,^ ^27^ Jaundice was also the most common clinical manifestation in a study conducted in the city of Salvador.^18^ It is worth noting that neonatal jaundice is common in all newborns (including those without congenital syphilis) and has several causes. Therefore, this finding should be interpreted with caution as to causality.

The lethality rate for congenital syphilis found in Santa Catarina State in the studied period was 8.5%, whereas a study carried out in Rio Grande do Sul State^26^ obtained a lethality rate of 3.6%, referring to the period from 2001 to 2012. This difference can be explained by the different periods analyzed in the two studies. However, it is noteworthy that the study conducted in Rio Grande do Sul had already found an increase in the lethality rate in the state: from 0.7%, in 2010, to 5.1%, in 2012. According to the Syphilis Bulletin, the death rate from congenital syphilis in Brazil was 6.1 per thousand live births in 2016, and the region with the highest rate was the Northeast (7.7 deaths per thousand live births). In the country, the infant mortality rate due to congenital syphilis increased from 2.3/100 thousand live births, in 2006, to 6.7/100 thousand live births, in 2016, and the Southern region showed considerable growth, as evidenced in the present study. For the analysis of deaths, only the SINAN records were used, with no consultation to the Mortality Information System (*Sistema de Informação de Mortalidade* - SIM) -SINAN may present flaws regarding the registration of deaths related to congenital syphilis.^28^

As study limitations, we highlight the lack of information in the notification forms regarding the clinical data of pregnant women and newborns, as well as many variables registered as “ignored”, which affects the analysis and interpretation of the reported cases. In addition, there is no follow-up of these newborns, because the notification form only corresponds to the period immediately after birth. However, this follow-up is recommended by the Ministry of Health, and should be performed by the services to monitor the response to treatment and the possible sequelae left by syphilis.^17^ Despite this, it is highlighted that it was possible to demonstrate the increase alarming number of cases of congenital syphilis in Santa Catarina State. The situational diagnosis of the present study can serve as planning for educational, preventive, diagnostic and therapeutic actions in cases of gestational and congenital syphilis.

It is observed that congenital syphilis remains a public health problem despite government efforts and measures. In 2018, the rapid response to syphilis project began to be implemented in the health care networks, which aims to eliminate congenital syphilis in Brazil. Congenital syphilis is a way of verifying the quality of maternal and child health care, having a relatively simple diagnosis and clinical and therapeutic management. Thus, the increased incidence of the disease indicates not only ineffective prenatal care, but also a failure in health education and access to services, and given that syphilis is a silent disease, laboratory screening tests are indicated by the Ministry Health and should be done routinely during prenatal care.
